# N6-methyladenosine (m6A) methyltransferase WTAP-mediated miR-92b-5p accelerates osteoarthritis progression

**DOI:** 10.1186/s12964-023-01228-8

**Published:** 2023-08-10

**Authors:** Zhaowei Lin, Tao Jiang, Wei Zheng, Jiayuan Zhang, Anan Li, Chao Lu, Wengang Liu

**Affiliations:** 1 Department of Joint and Orthopedics, Zhujiang Hospital of Southern Medical University, Guangzhou, 510000 China; 2 Orthopedics Department, Guangdong Provincial Second Hospital of Traditional Chinese Medicine, Guangzhou, 510095 China; 3 The Fifth Clinical College of Guangzhou University of Chinese Medicine, Guangzhou, 510405 China

## Abstract

The study was design to investigate the functional roles of Wilms tumor 1-associated protein (WTAP), an enzyme catalyzes m6A modification, in the pathogenesis of osteoarthritis (OA) and further elucidate its possible regulatory mechanism. Herein, we discovered that WTAP was outstandingly upregulated in chondrocyte stimulated with Lipopolysaccharide (LPS) and cartilage tissue of patients with OA. Functional studies have demonstrated that WTAP knockdown enhances proliferation ability, suppresses apoptosis, and reduces extracellular matrix (ECM) degradation in an LPS—induced OA chondrocyte injury model and ameliorates cartilage damage in a destabilizing the medial meniscus (DMM)—induced OA mice model. Conversely, overexpression of WTAP contributes to the opposite effects. Mechanistically, our data has demonstrated that m^6^A modification mediated by WTAP promotes the maturation of pri-miR-92b to miR-92b-5p, thereby enhancing the targeted inhibitory function of miR-92b-5p on TIMP4. Furthermore, we have discovered that WTAP can directly facilitate the degradation of TIMP4 mRNAs in a YTHDF2-dependent manner. In a nutshell, our findings suggested that WTAP knockdown alleviated OA progression by modulating the miR-92b-5p/TIMP4 axis in an m6A-dependent manner. Our study disclosed that WTAP-mediated m6A modification displayed a crucial role in OA development and suggested that targeting WTAP could be a promising preventive and therapeutic target for patients with OA.

Video Abstract

**Supplementary Information:**

The online version contains supplementary material available at 10.1186/s12964-023-01228-8.

## Introduction

Osteoarthritis (OA) is the most prevalent chronic joint disease in orthopaedics, characterized by cartilage loss as its primary lesion [1]. Patients with OA often experience joint pain, stiffness, and limited mobility, and even severe cases can progress to progressive disability, which greatly affects the their quality of life [2]. According to reports, approximately 80% of individuals over the age of 65 suffer from OA, especially knee OA [3]. To date, the management of OA remains a challenge because of its multifactorial etiology and evolving risk factors and pathophysiology [4]. The primary approach for OA treatment should involve non-pharmacological interventions complemented by pharmacotherapy, with surgical intervention reserved for severe case. In advanced stage, there is no effective treatment except joint replacement [5]. Therefore, elucidating the underlying mechanisms of OA may pave the way towards more precise therapeutic strategies.

Methylation of eukaryotic messenger RNA (mRNA) is essential for maintaining mRNA metabolism and function, thereby impacting cellular phenotype and function [6, 7]. N6-methyladenosine (m6A) modification, the insertion of a methyl substituent at the nitrogen atom at position 6 of adenosine, is the most prevalent internal RNA modification in mammalian cells and widely distributed in transcripts (> 25%) [8]. The m6A modification, unlike other RNA modifications, is dynamically reversible and mainly primarily regulates the maturation, translation and degradation of precursor mRNA through methyltransferases (METTL3, METTL14, and WTAP), demethylases (FTO and ALKBH5), and m6A methylated transcribed proteins (YTH domain family proteins, HNRNPA2B1 and IGF2BP) [9–11]. Therefore, imbalance of m6A affected by these proteins will affect the occurrence of various diseases, such as stem cell differentiation [12], leukemogenesis [13], osteoporosis [14], and cancers [11, 15], but its role and regulatory mechanism in OA are less studied.

In this study, we found that the WTAP was dramatically upregulated in OA cartilage tissue and chondrocytes. Functional studies demonstrated that WTAP knockdown suppressed LPS-induced chondrocyte injury in vitro and ameliorated OA cartilage damage in vivo. Conversely, overexpression of WTAP resulted in the opposite effects. Furthermore, we found that the inhibition of miR-92b-5p or TIMP4 significantly exacerbated and alleviated LPS-induced chondrocyte injury, respectively. Mechanistically, we revealed that WTAP regulated the miR-92b-5p/TIMP4 axis in an m6A-dependent manner. This study aimed to shed light on a new mechanism of OA progression, in which WTAP-mediated miR-92b maturation and TIMP4 mRNA degradation contribute to OA progression, and to suggest that WTAP may be a promising preventive and therapeutic target for OA.

## Materials and methods

### Cartilage specimens

OA cartilage tissue was gained from OA patients undergoing total knee replacement surgery, six of whom were used for primary chondrocyte culture. Control normal cartilage tissue was gained from patients with no previous history of OA undergoing amputation or trauma surgery. All clinical samples involved in this study were approved by the Ethics Committee of Guangdong Provincial Second Hospital of Traditional Chinese Medicine (No.2021-K59), and all participants signed written informed consent before the study.

### Cells culture

As previously described [16], primary chondrocyte was separated from articular cartilage gained from OA patient undergoing total knee replacement surgery (*n* = 6). Briefly, fresh articular cartilage tissues were collected and digested with 0.25% trypsin (Sigma-Aldrich). Following digestion at 37 °C for 30 min to remove fibroblasts, the articular cartilage was digested with 0.2% type II collagenase (Sigma-Aldrich) at 37 °C for 6 h and then filtered by a 100 μm cell strainer. Finally, the cells collected by centrifugation (1000 rpm/minutes, 10 min) were cultured in Dulbecco’s modified Eagle’s medium/nutrient mixture F12 (DMEM/F12, Thermo Fisher Scientific) supplemented 10% fetal bovine serum (FBS, Gibco) in the constant temperature incubator with 5% CO_2_. The cell morphology was observed under the microscope. The cells cultured to the second generation were identified as chondrocyte by immunofluorescence (collagen II antibody), in addition to observing the cells morphology, and the chondrocyte cultured to the third generation were used for later experimental research. The SW1353 cell line (a human chondrocyte-like cell line) was obtained from the American Type Culture Collection (ATCC, USA) and cultured in Dulbecco's Modified Eagle Medium (DMEM, Gibco) supplemented with 10% fetal bovine serum (Gibco BRL, Germany) in a humidified atmosphere with 5% CO_2_ at 37 °C.

### Adenovirus construction and cell transfection

The adenovirus for the overexpression / knockdown of WTAP, YTHDF2, and TIMP4 as well as their negative control were provided by Ribobio Inc. (Guangzhou, Guangdong, China). The chondrocyte was infected with the adenovirus according to the operating instructions, and the infection efficiency was determined by quantitative real-time reverse transcription PCR (qRT-PCR). The miR-92b-5p mimics, miR-92b-5p inhibitor, and their negative control were obtained from Genechem (Nanjing, China). The shRNA sequence, miR-92b-5p mimics sequence, miR-92b-5p inhibitor sequence, and their negative control sequence were listed in Table 1.

**Table 1:** All sequences of shRNAs, miR-92b-5p inhibitor/mimics, and their negative control used in this study

Names	Sequences (5’-3’)
shWTAP	CCGGATGGCAAGAGATGAGTTAATTCTCGAGAATTAACTCATCTCTTGCCATTTTTTGAATT
shYTHDF2	CCGGGATGGATTAAACGATGATGATCTCGAGATCATCATCGTTTAATCCATCTTTTTGAATT
shTIMP4	CCGGCCGGTATGAAATCAAACAGATCTCGAGATCTGTTTGATTTCATACCGGTTTTTGAATT
shNC	CCGGTCCTAAGGTTAAGTCGCCCTCGCTCGAGCGAGGGCGACTTAACCTTAGGTTTTTGAATT
miR-92b-5p mimics	sense, AGGGACGGGACGCGGUGCAGUG
	antisense, GGACGGGACGCGGUGCAGUGUU
NC mimics	sense, UUGUACUACACAAAAGUACUG
	antisense, GUACUUUUGUGUAGUACAAUU
miR-92b-5p inhibitor	CACTGCACCGCTCCCGTCCCT
NC inhibitor	CAGUACUUUUGUGUAGUACAA

### qRT-PCR

The cartilage tissue and chondrocytewere subjected to Trizol reagent (Beijing Jin Ming Biotechnology Co., Ltd., China) to extract the total RNA, and then total RNA was reverse transcribed into cDNA using PrimeScriptTM RT reagent Kit with gDNA Eraser (RR047A, Takara). RT-PCR amplification were performed on LightCycler®96 (Roche) using BenyoFastTMSYBR Green qPCR Mix (D7260, Beyotime) following the manufacturer protocols. U6 and GAPDH acted as internal control for miRNA and other genes, respectively. The expression levels of all genes were calculated through 2^−ΔΔCt^ method. The sequences of all primers were listed as in Table 2.

**Table 2:** All primer sequences used in the present study

Names	Primer sequences (5’-3’)
WTAP	Forward, CTTCCCAAGAAGGTTCGATTGA
	Reverse, TCAGACTCTCTTAGGCCAGTTAC
WTAP (mouse)	Forward, CTTCCGCGGACTGTCTCC
	Reverse, TCGTTGGTCATCTTGCACCC
YTHDF2	Forward, AGCCCCACTTCCTACCAGATG
	Reverse, TGAGAACTGTTATTTCCCCATGC
IGF2BP3	Forward, TCGAGGCGCTTTCAGGTAAA
	Reverse, TATCCAGCACCTCCCACTGTA
IGF2BP1	Forward, AAGGGGGCCATCGAGAATTG
	Reverse, CAGGGATCAGGTGAGACTGC
IGF2BP2	Forward, GGAACAAGTCAACACAGACACA
	Reverse, AACTGATGCCCGCTTAGCTT
ALKBH5	Forward, ACTGAGCACAGTCACGCTTCC
	Reverse, GCCGTCATCAACGACTACCAG
FTO	Forward, GAAGCACTGTGGAAGAAGATGGA
	Reverse, GGCAAGGATGGCAGTCAAGAT
RBM15	Forward, ATGCCTTCCCACCTTGTGAG
	Reverse, GGTCAGCGCCAAGTTTTCTC
YTHDF3	Forward, TGTTGTGGACTATAATGCGTATGC
	Reverse, AAGCGAATATGCCGTAATTGGTTA
YTHDF1	Forward, CAGCACCGATCCCGACATAG
	Reverse, CTGGCTTCCTGAAGACGATGA
TIMP4	Forward, GTGAAGATCGGACACTACGTG
	Reverse, CTGGAAGGTGGACAGCGAGG
pri-miR-92b	Forward, AGAGCCAGACACAGAAGA
	Reverse, GGACACGACCGTCCACCA
pre-miR-92b	Forward, AGGGAAAGGCGGAAGAGA
	Reverse, ATTGCCCTACCCGCCAAGAAATGTAAGGTAT
miR-92b-5p	Forward, ACACTCCAGCTGGG TTTAGTGTGATAATGGC
	Reverse, CAGTGCGTGTCGTGGAGT
GAPDH	Forward, CGGCAAGTTCAACGGCACAGT
	Reverse, ACGCCAGTAGACTCCACGACAT
GAPDH (mouse)	Forward, CCAGCTACTCGCGGCTTTAC
	Reverse, AATCCGTTCACACCGACCTT
U6	Forward, CTCGCTTCGGCAGCACA
	Reverse, TGGTGTCGTGGAGTCG
Primers used for MeRIP-qPCR	
Pri-miR-92b	Forward, CTTCTGGGACTCCGCAAACT
	Reverse, TTGGAGGCCAGAGAGACTTG
TIMP4	Forward, AACAGCCAGAAGCAGTATC
	Reverse, TGTGTAGCAGGTGGTGAT
Primiers used for in vitro transcription	
Pri-miR-92b	Forward, CCGCTCGAGTAATACGACTCACTATAGGGAGAAGTCTGAGTACTT AAAGAGCAAGCGC
	Reverse, CTAGTCTAGATAGAAGAGAAAGCCTGGGAGGGT
Pri-miR-1–1	Forward, CCGCTCGAGTAATACGACTCACTATAGGGAGAAAGGCTGTCCTGC TCACACA
	Reverse, CTAGTCTAGATCCCGGCCTGAGATACATAC

### Western blot

The cartilage tissue and chondrocytewere exposed to RIPA lysis buffer (Solarbio) to extract total protein, followed by quantification using bicinchoninic acid (BCA) detection kit (Shanghai UCHEM Inc., China). Afterwards, the separated proteins were transferred onto PVDF membranes (Zhejiang Lianshuo Biotechnology Co., Ltd., China). After blocking with 5% non-fat milk for 2 h, the membranes were incubated overnight at 4℃ with primary antibody, followed by incubated with anti-rabbit IgG (ab14708, 1:5000, Abcam) or anti-mouse IgG (ab3420, 1:5000, Abcam) at room temperature for 60 min. Finally, bands were visualized on gel imaging analysis system (Bio-Rad) using ECL chemiluminescence kit (Beyotime, P0018AS) and quantified using an Image J software (National Institutes of Health, Bethesda, Maryland, USA). The primary antibodies were as follows: anti-WTAP (ab195380, 1:1000, Abcam), anti-ADAMTS5 (ab41037, 1:250, Abcam), anti-MMP13 (ab39012, 1:3000, Abcam), anti-Collagen II (ab34712, 1:1000, Abcam), anti-Aggrecan (ab3778, 1:750, Abcam), anti-TIMP4 (ab58425, 1:750, Abcam), and anti-GADPH (ab8245, 1:1000, Abcam).

### Total RNA m6A quantification

Total RNA was separated using TRIzol reagent (Takara, Japan), and then m6A level of total RNA was quantified through m6A RNA methylation assay kit (colorimetric; Abcam, ab185912) in the light of the instructions. Briefly, 200 ng of purified RNA was coated with a trapping antibody solution at a suitable dilution concentration. The absorbance was detected using a microplate reader (Tecan, F50) with the wavelength of 450 nm, and the relative m6A levels were computed by the following formula: m6A% = [(Sample OD-NC OD)/S] × 100%/[(PC OD-NC OD)/P], where the OD indicates absorbance value at 450 nm, NC indicates negative control, PC indicates positive control, S indicates the amount of input sample RNA, and P indicates the amount of input positive control.

### Proliferation analysis

Proliferation was evaluated by the cell counting kit-8 (CCK-8) assay and a 5-ethynyl-20-deoxyuridine (EdU) assay. For CCK-8 assay, the treated cells were dealt with enhanced cell counting kit-8 (Beyotime, C0042) according to the instructions. The absorbance of each sample was conducted by a microplate reader (Tecan, F50) at 450 nm. For EdU assay, the treated cells were conducted by EdU assay kit (Ribobio, Guangzhou, China) on the basis of the instructions. After nuclear staining using Hoechst 33,342, the images were obtained by an inverted fluorescence microscope (Mshot, MF52) and then calculated using Image J software (National Institutes of Health, Bethesda, Maryland, USA).

### Apoptosis analysis

The Annexin V-FITC Apoptosis Detection Kit (Beyotime, C1062L) and the Caspase-3 Colorimetric Assay Kit (Keygen Biotech, Nanjing, China) were utilized to determine cell apoptosis. For the Annexin V-FITC Apoptosis Detection Kit, the treated chondrocyte was suspended in the binding buffer containing Annexin V and incubated for 20 min. Afterwards, the chondrocyte was incubated with PI for 15 min. The percentage of chondrocyte apoptosis was examined by a FACScan flow cytometer (Becton Dickinson, USA). In addition, caspase-3 activity was detected using the Caspase-3 Colorimetric Assay Kit (Biovision, K106-25). The absorbance of each sample was measured by a microplate reader (Tecan, F50) at 450 nm.

### MiRNA sequencing and data analysis

The miRNA sequencing and data analysis were executed by Shanghai Biotechnology Corporation (Shanghai, China). In short, total RNAs were separated from each sample using mirVana™ miRNA Isolation Kit (Cat #. AM1561, Austin TX, US) following the manufacturer's standard operating procedures, and then got through the electrophoresis quality inspection by Agilent 2100 Bioanalyzer (Agilent Technologies, Santa Clara, USA) for subsequent detection. Then, the sequencing sample library construction and cluster generation were performed. After the sequencing sample library construction and cluster generation were completed, the flow cell carrying the cluster was sequenced on the machine. The sequencing process was commanded by the data collection software provided by Illumina, and real-time data analysis was carried out. The differentially expressed miRNAs were identified with P < 0.05 and |log_2_fold-change (FC)|> 2.

### Data sources and bioinformatics analysis

A sequence-based N6-methyladenosine (m6A) modification site predictor (SRAMP) was used to predict m6A modification sites on pri-miR-92b and TIMP4. The potential targets of miR-92b-5p were forecast through TargetScan (http://www.targetscan.org/), and then these predicted targets were crossed with significantly downregulated genes (FC < -4 and *P*-value < 0.01) in OA cartilage tissue derived from GSE113825, which was obtained from Gene Expression Omnibus (GEO) database (https://www.ncbi.nlm.nih.gov/geo/query/acc.cgi).

### Methylated RNA immunoprecipitation (MeRIP)-qPCR

The chondrocyte was carried out using the Magna MeRIP™ m6A Kit (Beijing Baiao Innovation Technology Co., Ltd., China). In short, total RNA isolated from chondrocyte was subjected to fragment, then immunoprecipitated with m6A antibody (ab286164; Abcam) or anti-mouse IgG (ab6715, Abcam) conjugated with protein A/G magnetic beads (Cell Signaling Technology). After precipitation, RNA was eluted from the beads and purified, and the purified RNA was subsequently used to perform qRT-PCR.

### In vitropri-miRNA processing assays

In vitro assays for pri-miR-92b processing was conducted following previously reported methods [17, 18]. In brief, the mutant pri-miR-92b[m^6^A] was generated by replacing adenosine (A) with guanine (G). Subsequently, pri-miR-92b[m^6^A]-WT or pri-miR-92b[m^6^A]-Mut and pri-miR-1–1 (control) were incubated with whole cellular lysates of SW1353 cells co-overexpressing DROSHA and DGCR8. Finally, total RNA extracted from reaction products was subjected to qRT-PCR analysis.

### RNA immunoprecipitation (RIP)

The EZ-Magna RIP™ RNA-Binding Protein Immunoprecipitation Kit (Millipore) was applied to conduct the RIP experiment. Briefly, the treated cells were lysed using RIP lysis buffer, and then incubated with magnetic beads coupled with the anti-Ago2 antibody (ab186733, Abcam), anti-DGCR8 antibody (ab191875, Abcam), or IgG antibody (ab6715, Abcam) at 4℃. Following washing, the co-precipitated RNAs were extracted from magnetic bead complex using the TRIzol reagent (Takara, Japan), after which qRT-PCR was performed utilizing specific primers.

### Luciferase reporter assay

To investigate the effect of m6A modification on pri-miR-92b or TIMP4 expression, we employed SRAMP to predict potential m^6^A modification sites on pri-miR-92b and TIMP4 sequences. Based on these predictions, mutagenesis from A to G was generated by QuikChange II Site-Directed Mutagenesis Kit (Agilent, USA) in the light of the instruction. Subsequently, the wild-type and m6A mutant pri-miR-92b or TIMP4 reporter vectors was co-transfected with shWTAP or WTAP plasmid into SW1353 cells. To confirm the targeted interaction between miR-92-5p and TIMP4, the wild type or mutant type of TIMP4 3′-UTR (containing the binding site of miR-92b-5p) was cloned into the luciferase vector, and then transfected into SW1353 cells together with miR-92b-5p mimics or NC mimics by Lipofectamine 3000 (Invitrogen, USA), respectively. After 48 h of transfection, the luciferase activity was assessed using a Dual Luciferase Assay Kit (Promega, Madison, WI, USA) according to the instructions. Finally, the signal value of renilla luciferase was normalized as an internal reference.

### RNA decay assay

RNA stability was evaluated through an RNA decay assay. In short, chondrocytealone or co-transfected with WTAP and shYTHDF2 were treated with 5 μg/mL actinomycin D (MCE, HY-17559). Chondrocyte was collected at 0, 1, 3, and 5 h post-treatment, and then total RNA was extracted for qRT-PCR analysis to determine the relative abundance of TIMP4 mRNA.

### Destabilizing the medial meniscus (DMM)-induced OA mouse model

All animal experiments were executed in the light of the Guide for the Care and Use of Laboratory Animals and confirmed by the Animal Care Committee of Guangdong Provincial Second Hospital of Traditional Chinese Medicine (No: *048912). The male C57BL/6 mice (SPF, Eight-week-old) were randomly divided into four groups: sham (*n* = 6), model (*n* = 6), model + adeno-associated virus-negative control** (**AVV-shNC**)** (*n* = 6), and model + AVV-shWTAP groups (*n* = 6). After acclimating for one week, mice in other groups were treated with DMM surgery to induce OA as previously described except for the sham groups [19]. The mice in sham group were underwent only the skin of the right knee joint incision. The AAV-shNC and AAV-shWTAP were constructed by HANBIO (Shanghai, China). The AAV-shNC and AAV-shWTAP groups were intra-articularly injected with 10 μL of AAV-shNC and AAV-shWTAP (1 × 10^13^ vg/ml) through the medial parapatellar area at two weeks after the DMM operation, respectively. At the same point in time, the mice in sham and model groups were intra-articularly injected with an equal volume of normal saline. Eight weeks after surgery, knee joints were harvested from euthanized mice for later histological analysis and molecular analysis.

### Histological analysis

Eight weeks after modeling, the C57BL/6 mice were euthanized and the harvested knee joints were stained with hematoxylin–eosin (H&E) and safranin O/fast green for histological analysis. Briefly, the freshly harvested knee joints were fixed with 4% paraformaldehyde, decalcified in 15% EDTA-2Na, dehydrated in gradient ethanol, embedded in paraffin, and then sliced into 5-μm thick sections. Following dewaxing in xylene and hydration with a graded ethanol series, the sections were stained with H&E (Sigma-Aldrich) or safranin O/fast green (Shanghai Wowu Biological Technology Co., Ltd., China), respectively. The histopathological changes in cartilage tissues were visualized and photographed under Orthotopic light microscope (Leica, Cat. DMI1). Additionally, the degree of articular cartilage damage was evaluated using Osteoarthritis Research Society International (OARSI) score, as previously described [20].

### Statistical analysis

All data were shown as mean ± standard deviation (SD). The statistical analysis was performed using GraphPad Prism 8.0 and the differences were analyzed using Student’s t-test and one-way ANOVA assay. Each research had at least three biological replicates and the *P*-value < 0.05 indicated a statistically significant difference.

## Results

### WTAP was elevated in OA and contributed to increased m6A levels

**Fig. 1:**
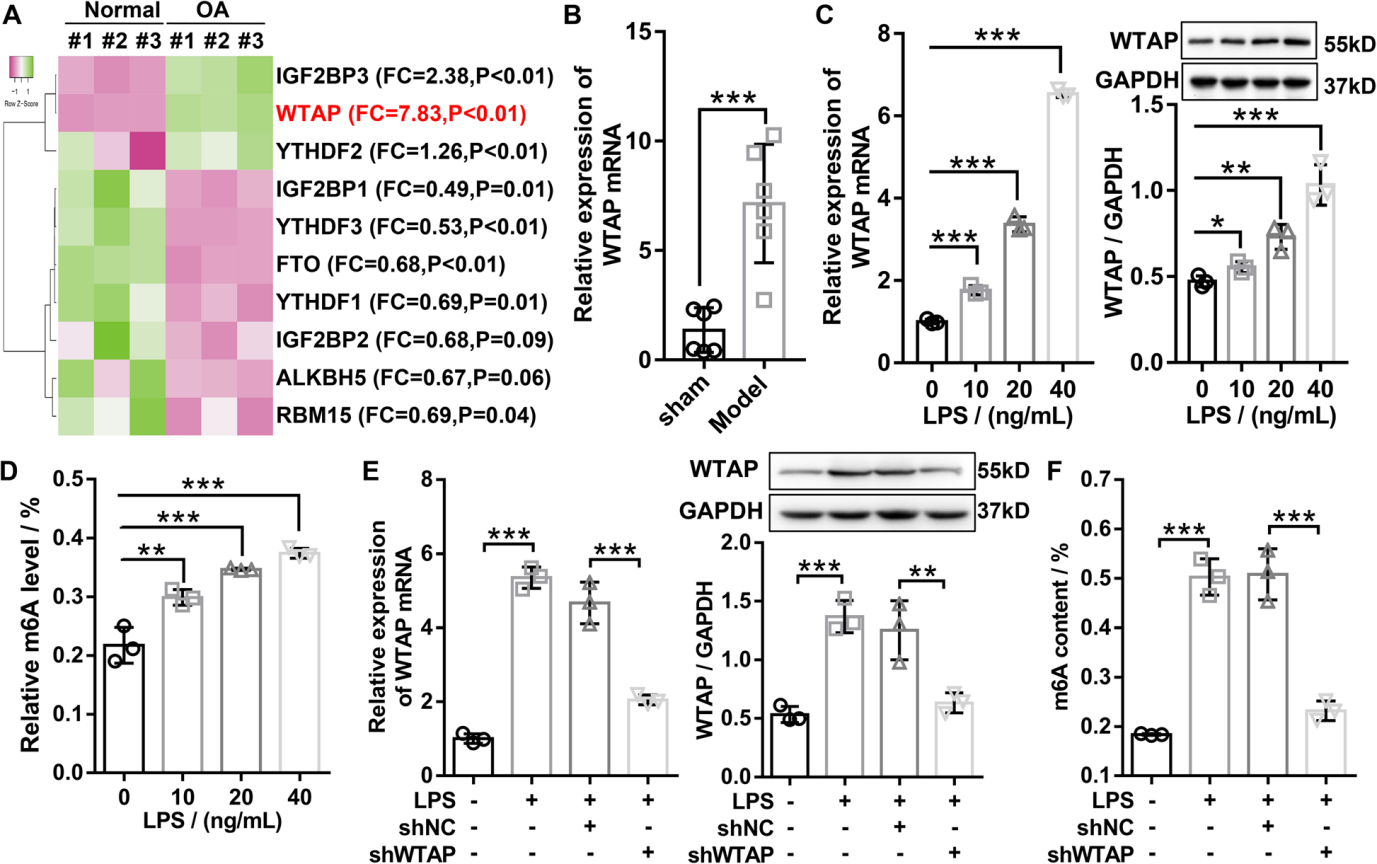
WTAP was elevated in OA and contributed to increased m6A levels. **A** Heat map showed that the mRNA expression of m6A modification-related genes in human OA cartilage tissue and human normal cartilage tissue was detected by qRT-PCR. **B** The mRNA expression of WTAP in mice OA cartilage tissue and mice normal cartilage tissue (*N* = 6) was detected by qRT-PCR. **C** The WTAP expression were measured by qRT-PCR and western blotting, and the results indicated that WTAP significantly increased in LPS-induced OA chondrocytes in a dose-dependent manner. **D** Total m6A levels were measured by m6A RNA methylation assay kit, and the results indicated that total m6A levels was increased in LPS-induced OA chondrocyte in a dose-dependent manner. **E** RT-qPCR and western blotting were applied to confirm the inhibition of WTAP in LPS-induced OA chondrocyte. **F** WTAP knockdown significantly suppressed LPS-induced total m6A levels in chondrocyte. OA, osteoarthritis; Sham, mice without destabilizing the medial meniscus (DMM); model, DMM surgery-induced OA mice; LPS, lipopolysaccharide; shWTAP, WTAP knockdown adenovirus; shNC, negative control corresponding to shWTAP. *N* = 3 ~ 6. ^*^P < 0.05, ^**^P < 0.01, and.^***^P < 0.001

To investigate the role of m6A modification in OA, we performed qRT-PCR to measure the mRNA expression of m6A modification-related enzymes in human OA cartilage tissue (*N* = 3) and human normal cartilage tissue (*N* = 3). Compared with normal cartilage tissue, three m6A modification-related enzymes were markedly up-regulated and five m6A modification-related enzymes were significantly down-regulated in OA cartilage tissue, among which WTAP had the most significant difference (Fig. 1A). Consistently, WTAP was found to be sharply up-regulated in both OA animal models and LPS-induced OA cell models (Fig. 1B and C). Meanwhile, quantitative analysis of the m6A modifications revealed that m6A levels in the total RNA were significantly elevated in an LPS concentration-dependent manner (Fig. 1D). WTAP knockdown was further confirmed by western blotting and qRT-PCR in LSP-induced OA chondrocytes (Fig. 1E), and found that LPS-induced m6A levels was remarkably suppressed by WTAP knockdown (Fig. 1F). Collectively, the findings implied that WTAP was outstandingly upregulated in OA cartilage tissues and LPS-induced OA chondrocytes, and WTAP knockdown obviously suppressed overall levels of m6A modification in OA.

### WTAP knockdown promoted proliferation and ECM synthesis, as well as inhibited apoptosis in LPS-inudced OA chondrocyte

**Fig. 2:**
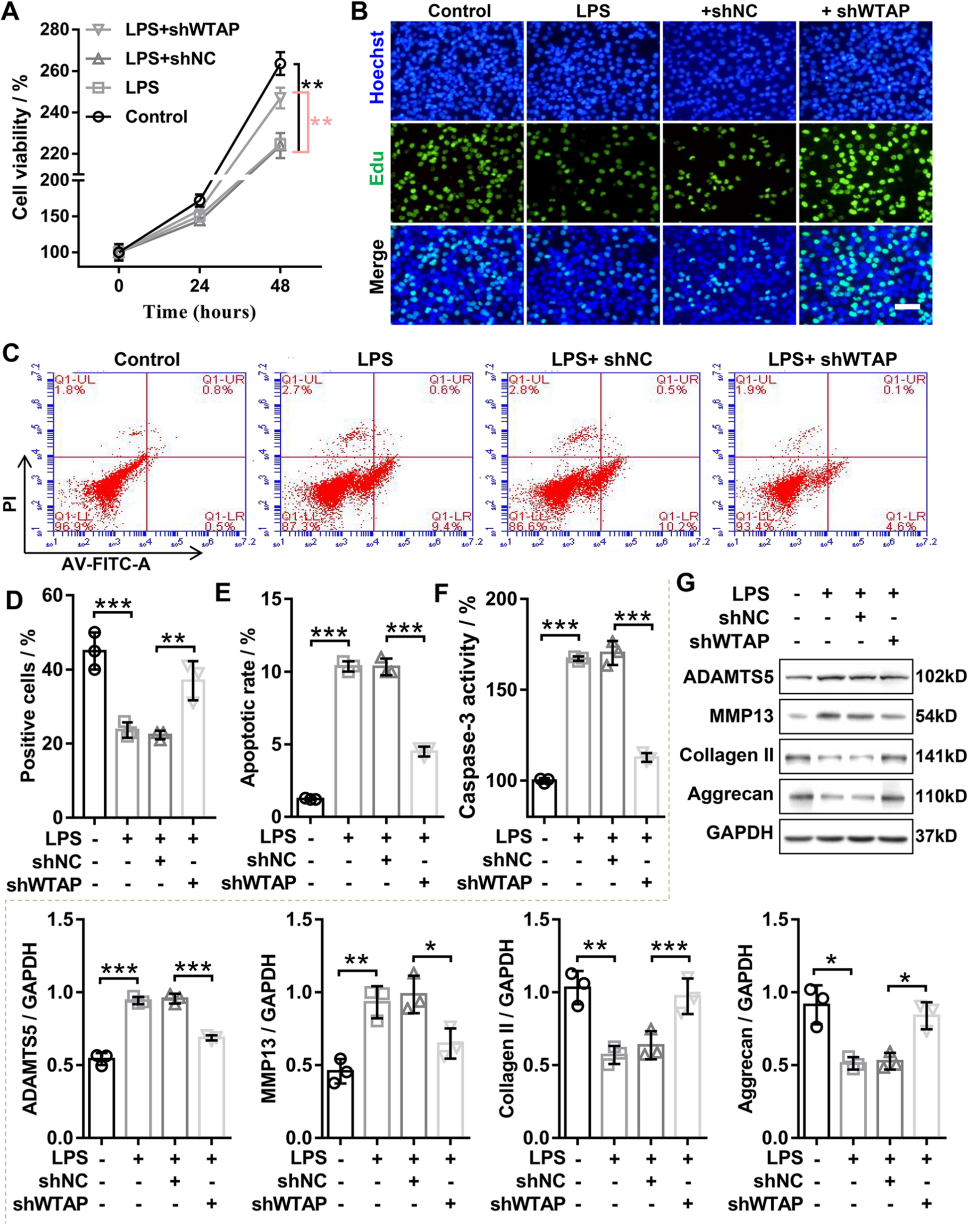
WTAP knockdown promoted proliferation and ECM synthesis as well as inhibited ECM degradation and apoptosis in LPS-induced OA chondrocyte injury. Chondrocyte was infected with shWTAP or shNC, and then treated with 40 ng/mL LPS for 24 h or 48 h. **A** CCK-8 was applied to evaluate the viability of chondrocyte. **B** and **D** EdU staining was used to measure the proliferation of chondrocyte. **C** and **E** The apoptosis of chondrocyte was evaluated by flow cytometry with Annexin V-FITC Apoptosis Detection Kit. **F** The caspase-3 activity was evaluated by caspase-3 activity kit. **G** Western blotting was used to assess the protein expression levels of MMP13, ADAMTS5, Aggrecan, and Collagen II in chondrocyte. OA, osteoarthritis; LPS, lipopolysaccharide; shWTAP, WTAP knockdown adenovirus; shNC, negative control corresponding to shWTAP. *N* = 3. ^*^P < 0.05, ^**^P < 0.01, ^***^P < 0.001

**Fig. 3:**
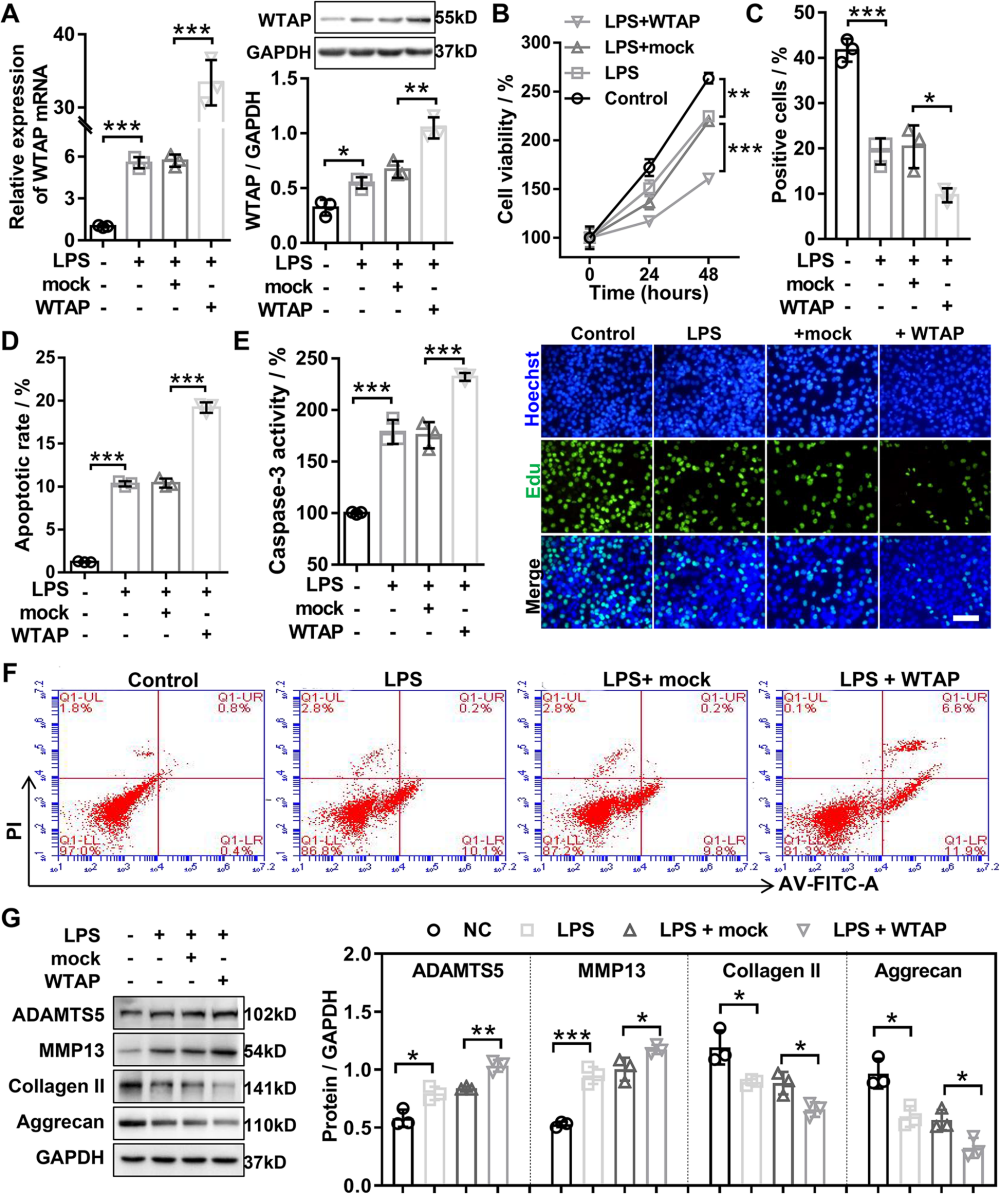
WTAP overexpression inhibited proliferation and ECM synthesis as well as promoted ECM degradation and cell apoptosis in LPS-induced OA chondrocyte injury. Chondrocyte was infected with WTAP or mock, and then treated with 40 ng/mL LPS for 24 h or 48 h. **A** The qRT-PCR and western blotting were applied to confirm the overexpression of WTAP in LPS-induced OA chondrocyte. **B** CCK-8 was applied to evaluate the viability of chondrocyte. **C** EdU staining was applied to detect the proliferation of chondrocyte. **D** and **F** The apoptosis of chondrocyte was evaluated by flow cytometry with Annexin V-FITC Apoptosis Detection Kit. **E** The caspase-3 activity was evaluated by caspase-3 activity kit. **G** Western blotting was applied to assess the protein expression levels of MMP13, ADAMTS5, Aggrecan, and Collagen II in chondrocyte. OA, osteoarthritis; LPS, lipopolysaccharide; WTAP, WTAP overexpression adenovirus; mock, negative control corresponding to WTAP. *N* = 3. ^*^P < 0.05, ^**^P < 0.01, and.^***^P < 0.001

To explore the biological function of WTAP on OA chondrocyte, we performed gain- and loss-of-function analysis of WTAP in LPS-induced OA chondrocyte. We constructed the overexpression adenovirus of WTAP (WTAP) and the RNA interference adenovirus of WTAP (shWTAP), and the results of qRT-PCR and western blotting disclosed that the mRNA and protein expression of WTAP was upregulated and inhibited in LPS-induced OA chondrocyte transfected with WTAP and shWTAP, respectively (Figs. 1E and 3A). Growth curves performed by CCK8 assays showed that WTAP knockdown partly mitigated LPS-induced proliferation inhibition in chondrocyte (Fig. 2A), whereas upregulation of WTAP obviously aggravated the inhibition of chondrocyte proliferation by LPS (Fig. 3B). Similarly, EdU assay uncovered that WTAP knockdown dramatically increased the percentage of EdU-positive cells in LPS-induced OA chondrocyte (Fig. 2B and D), while overexpression of WTAP displayed an opposite effect (Fig. 3C). The flow cytometry and caspase-3 activity assays were applied to examine the effects of WTAP on LPS-induced OA chondrocyte apoptosis. The results of flow cytometry showed that WTAP knockdown significantly inhibited the LPS-induced OA chondrocyte apoptosis (Fig. 2C and E), while overexpression of WTAP markedly exacerbated LPS-induced OA chondrocyte apoptosis (Fig. 3D and F). Consistently, we found that WTAP knockdown dramatically reduced LPS-induced caspase-3 activity in chondrocyte (Fig. 2F), and yet overexpression of WTAP exhibited an opposite effect (Fig. 3E). The balance of extracellular matrix (ECM) synthesis and degradation was an important factor in maintaining cartilage regeneration, western blotting was applied to evaluate the protein expression of ECM anabolic markers (Collagen II and Aggrecan) and ECM catabolic markers (ADAMTS5 and MMP13). The results depicted that knockdown of WTAP in LPS-induced OA chondrocyte resulted in significant downregulation of ADAMTS5 and MMP13 protein levels and significant upregulation of collagen II and Aggrecan protein levels (Fig. 2G). On the contrary, overexpression of WTAP significantly accelerated the upregulation of ADAMTS5 and MMP13 as well as outstandingly promoted the repression of collagen II and Aggrecan in LPS-induced OA chondrocyte (Fig. 3G). Overall, knockdown of WTAP inhibited LPS-inudced OA chondrocyte injury, and overexpression of WTAP aggravated LPS-inudced OA chondrocyte injury in vitro.

### WTAP knockdown inhibited OA deterioration in vivo

**Fig. 4:**
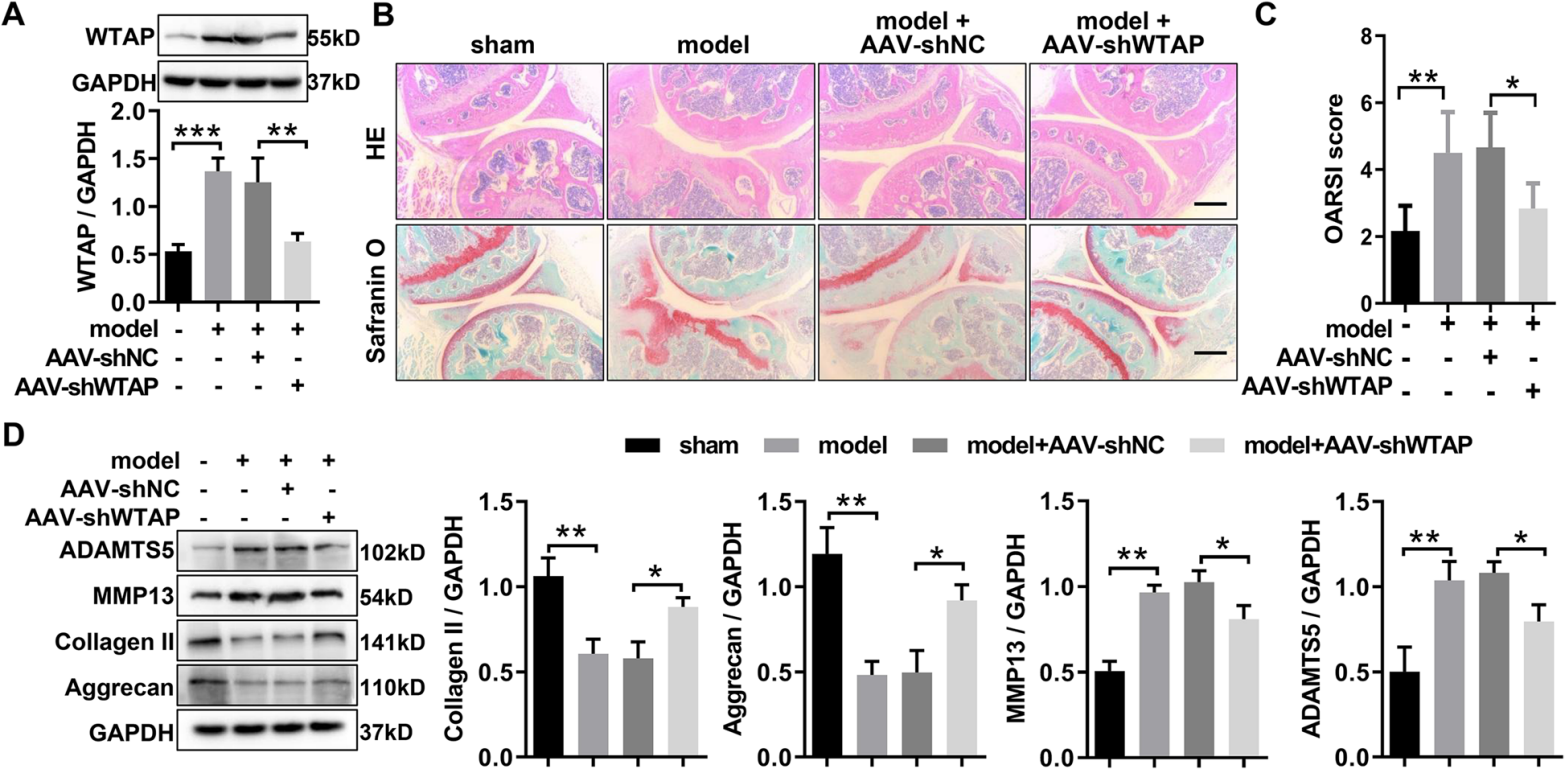
WTAP knockdown ameliorated the OA progression in mice. **A** The protein expression of WTAP was evaluated by western blotting in the knee cartilage of OA mice with or without AAV-shWTAP treatment and sham mice. **B** The section of the knee joints was stained with H&E and Safranin O & Fast Green, respectively. **C** OARSI scores of the knee joints in OA mice with or without AAV-shWTAP treatment. **D** The protein expression levels of MMP13, ADAMTS5, Aggrecan, and Collagen II were evaluated by western blotting in the knee cartilage of OA mice with or without AAV-shWTAP treatment. OA, osteoarthritis; Sham, mice without destabilizing the medial meniscus (DMM); model, DMM surgery-induced OA mice; AAV- shWTAP, WTAP knockdown adeno-associated virus; AAV-shNC, negative control corresponding to AAV-shWTAP. *N* = 3 ~ 6. ^*^P < 0.05, ^**^P < 0.01, and ^***^P < 0.001

To investigate the impact of WTAP on OA progression in vivo, the DMM-induced OA mice were injected intra-articularly with AAV-shWTAP or AAV-shNC. Western blotting was used to ascertain the efficiency of WTAP knockdown after AAV injection in cartilage tissue. This result disclosed that the WTAP was outstandingly downregulated in articular cartilage of OA mice compared with that of in articular cartilage of sham mice, and AAV-shWTAP treatment significantly decreased WTAP expression in articular cartilage of OA mice (Fig. 4A). We then used the H&E and Safranin O/fast green to investigate histopathological changes in cartilage surface after intra-articular injection of AAV-shWTAP in OA mice. The results showed that OA mice showed more severe degenerative OA changes than the sham mice, and these modifications were obviously ameliorated following AAV-shWTAP treatment (Fig. 4B). Consistent with histological analyses, OARSI scores in articular cartilage of OA mice prominently increased compared with that in articular cartilage of sham mice, but the increased OARSI scores were obviously inhibited following AAV- shWTAP treatment (Fig. 4C). Finally, western blotting was applied to estimate the protein expression of ECM-related markers (MMP-13, ADAMTS, Collagen II, and Aggrecan) in articular cartilage and found that AAV-shWTAP treatment obviously suppressed the expression of MMP-13 and ADAMTS-5 as well as outstandingly augmented the expression of Collagen II and Aggrecan in articular cartilage of OA mice (Fig. 4D). Taken together, these results confirmed that WTAP knockdown suppressed OA progression, suggesting that targeting WTAP may be a promising therapeutic strategy for OA.

### WTAP mediated miR-92b-5p maturation in an m6A-dependent manner

**Fig. 5:**
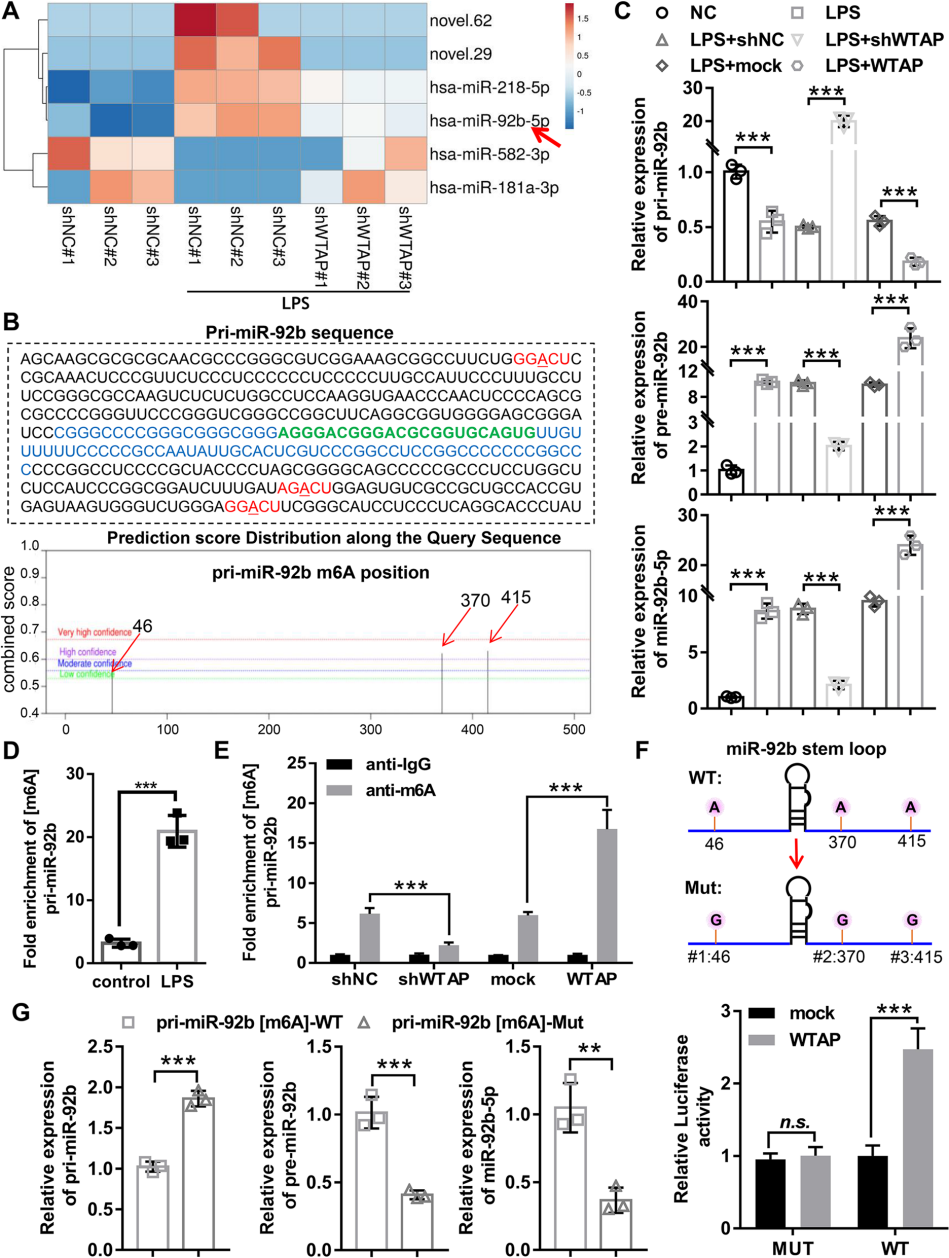
WTAP mediated miR-92b-5p maturation in an m6A-dependent manner. **A** The heatmap showed that differential miRNAs expression profiles obtained by miRNA-Seq analysis between the shNC, LPS + shNC, and LPS + shWTAP groups. **B** The sequence of pri-miR-92b and the potential m6A site of pri-miR-92b. Green indicated miR-92b-5p sequence, blue indicated pre-miR-92b sequence, and red indicated m6A sites. **C** Chondrocyte was infected with shWTAP, shNC, mock, or WTAP, and then dealt with 40 ng/mL LPS for 48 h. The qRT-PCR was used to assess the RNA expression of pri-miR-92b, pre-miR-92b, and miR-92b-5p. **D** MeRIP coupled with qRT-PCR was used to detect the m6A levels of pri-miR-92b in chondrocyte with or without LPS. **E** Chondrocyte was infected with shWTAP, shNC, mock, or WTAP, and then the m6A levels of pri-miR-92b were evaluated using MeRIP coupled with qRT-PCR. **F** Relative luciferase activity of the wild-type or mutant-type of [m6A] pri-miR-92b reporter vectors was assessed in WTAP-overexpression chondrocyte. **G** Quantification of miR-92b-5p, pre-miR-92b, and pri-miR-92b in the reaction mixture was detected by qRT-PCR, and found that mutation of [m6A] pri-miR-92b abolished pri-miR-92b processing in the in vitro reaction system. LPS, lipopolysaccharide; WTAP, WTAP overexpression adenovirus; mock, negative control corresponding to WTAP; shWTAP, WTAP knockdown adenovirus; shNC, negative control corresponding to shWTAP. *N* = 3. ^n.s.^*P* > 0.05, ^**^P < 0.01, and ^***^P < 0.001

To investigate the molecular mechanism of WTAP in OA cartilage injury, we identified WTAP-regulated miRNAs using miRNA-sequencing analysis. The miRNAs expression profiling revealed that four significantly upregulated miRNAs (novel.29, novel.62, hsa-miR-218-5p, and hsa-miR-92b-5p) and two markedly downregulated miRNAs (hsa-miR-582-3p and hsa-miR-181a-3p) in LPS-induced OA chondrocyte were reversed by WTAP knockdown (Fig. 5A). Since the increased and decreased m6A levels lead to the promote and arrest of pri-miRNA processing, we selected miRNAs (hsa-miR-218-5p and hsa-miR-92b-5p) regulated by WTAP to predict m^6^A modification sites on pri-miR-92b and pri-miR-218 sequence using SRAMP. The results showed that 3 m6A motif in pri-miR-92b RNA sequence (Fig. 5B), whereas the m6A motif site was not found in pri-miR-218 RNA sequence. Therefore, we hypothesized that WTAP might mediate the maturation of pri-miR-92b. We first examined the expression of pri-miR-92b, pre-miR-92b and miR-92b-5p in chondrocyte, and found that WTAP knockdown sharply increased the accumulation of pri-miR-92b but decreased the expression of both pre-miR-92b and miR-92b-5p in LPS-induced OA chondrocytes, while WTAP overexpression displayed opposite effect (Fig. 5C), suggesting that the miR-92b-5p induction by LPS may be through WTAP-mediated aberrant pri-miR-92b processing and maturation. The miR-92b-5p expression levels in normal and OA cartilage tissue were measured by RT-qPCR, and found that the expression levels of miR-92b-5p in human OA cartilage tissue were significantly higher than those in human normal cartilage tissue (Additional file 1: Fig. S1A). Meanwhile, we also found that knockdown of WTAP significantly inhibited the high expression of miR-92b-5p in cartilage tissue of OA mice (Additional file 1: Fig. S1B). The above results implied that WTAP-mediated m6A modification might facilitate the processing of pri-miR-92b and miR-92b-5p maturation.

MeRIP coupled with qRT-PCR displayed that the pri-miR-92b m6A levels were significantly elevated in LPS-induced OA chondrocyte compared with control (Fig. 5D). Furthermore, MeRIP coupled with qRT-PCR confirmed that overexpression of WTAP dramatically enhanced pri-miR-92b m6A levels in LPS-induced OA chondrocyte, but WTAP knockdown remarkably decreased pri-miR-92b m6A levels in LPS-induced OA chondrocyte (Fig. 5E). Luciferase reporter assay showed that WTAP overexpression moderately enhanced the luciferase activity of the pri-miR-92 [m^6^A]-WT but had not affect the luciferase activity of the pri-miR-92 [m^6^A]-MUT (Fig. 5F). These data demonstrated that WTAP-mediated m6A modification was involved in the maturation of miR-92b-5p. To further confirm a direct function of m^6^A in the maturation of miR-92b-5p, we performed in vitro pri-miRNA processing assays. The results indicated that the processing rate of pri-miR-92b to pre-miR-92b and miR-92b-5p in pri-miR-92b[m^6^A]-Mut was outstandingly decreased compared with that of in pri-miR-92b [m^6^A]-WT (Fig. 5G). Collectively, our data demonstrated that WTAP promoted the maturation of miR-92b-5p in an m^6^A-dependent manner.

### TIMP4 was the target of miR-92b-5p, and TIMP4 inhibition reversed the effects of miR-92b-5p knockdown on proliferation, apoptosis, and ECM degradation in LPS-induced OA chondrocyte

**Fig. 6:**
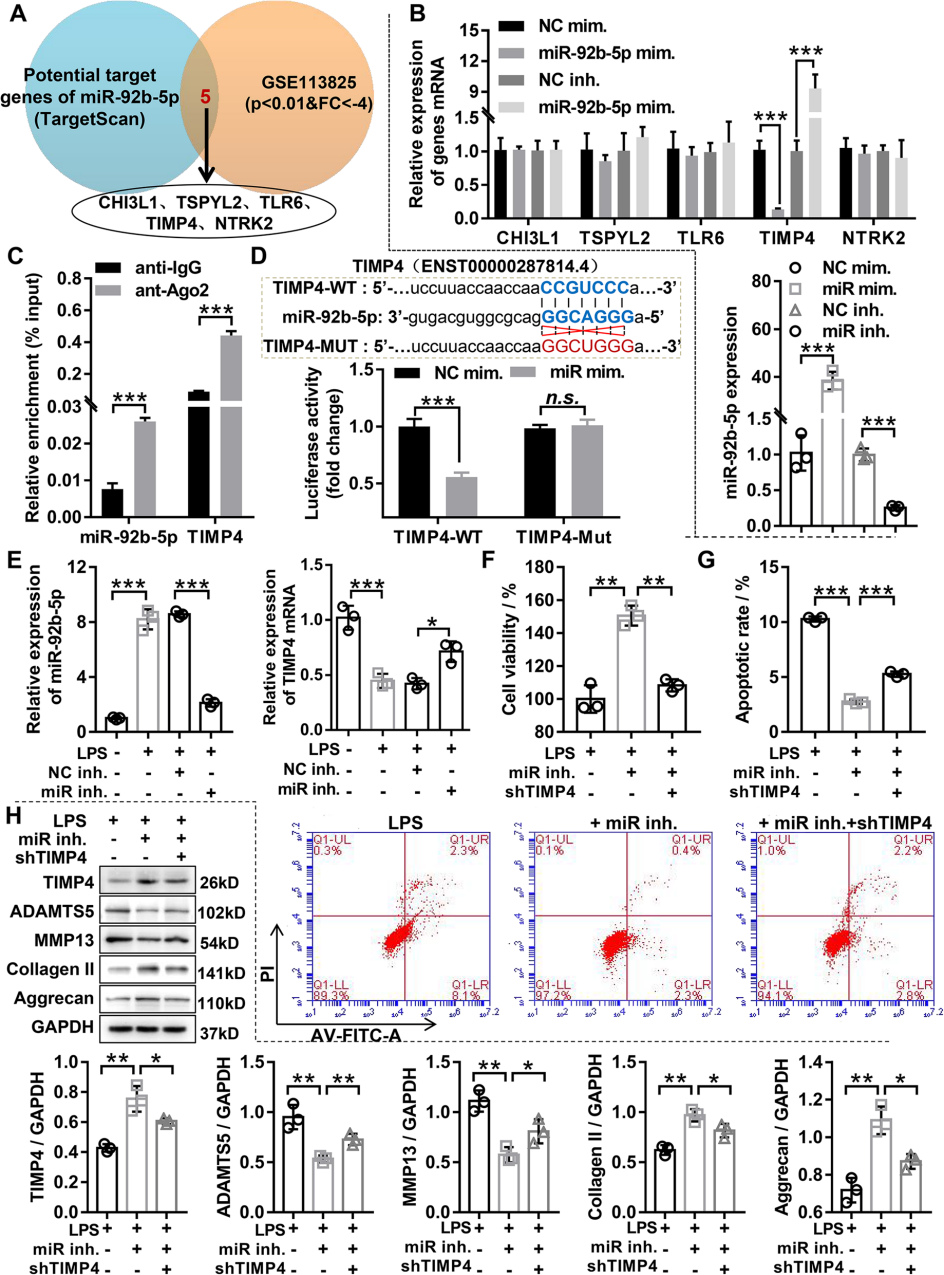
TIMP4 was a target of miR-92b-5p, and TIMP4 inhibition reversed the effects of miR-92b-5p knockdown on proliferation, apoptosis, and ECM degradation in LPS-induced OA chondrocytes. **A** The target genes predicted by TargetScan for miR-92b-5p were intersected with the significantly down-regulated mRNAs in OA cartilage from GSE113825. **B** The mRNAs expression of these target genes was tested by qRT-PCR in chondrocyte with miR-92b-5p overexpression or knockdown. **C** RIP-Ago2 analysis indicated binding of miR-92b-5p to TIMP4. **D** Interaction between miR-92b-5p and TIMP4 was affirmed by dual luciferase reporter assay. **E** Chondrocyte transfected with miR-92b-5p inhibitor or NC inhibitor was treated with 40 ng/mL LPS for 48 h. RT-qPCR was used to detect the mRNA expression of miR-92b-5p and TIMP4. **F** Chondrocyte was transfected with miR-92b-5p inhibitor alone or in combination with shTIMP4, and then treated with 40 ng/mL LPS for 48 h. CCK-8 was detected the viability of chondrocyte. **G** Chondrocyte was transfected with miR-92b-5p inhibitor alone or in combination with shTIMP4, and then treated with 40 ng/mL LPS for 48 h. The apoptosis of chondrocyte was tested by flow cytometry. **H** Chondrocyte was transfected with miR-92b-5p inhibitor alone or in combination with shTIMP4, and then treated with 40 ng/mL LPS for 48 h. Western blotting was applied to assess the protein expression of MMP13, ADAMTS5, Aggrecan, and Collagen II. LPS, lipopolysaccharide; NC mim., negative control corresponding to miR-92b-5p mimics; miR mim., miR-92b-5p mimics; NC inh., negative control corresponding to miR-92b-5p inhibitor; miR inh., miR-92b-5p inhibitor; shTIMP4, TIMP4 knockdown adenovirus. *N* = 3. ^n.s.^*P* > 0.05, ^*^P < 0.05, ^**^P < 0.01, and.^***^P < 0.001

To explore the regulatory mechanism of miR-92b-5p in OA, the target mRNAs predicted by TargetScan for miR-92b-5p were intersected with the significantly downregulated mRNAs from GSE113825 in OA cartilage tissue. The results found that miR-92b-5p has five potential target genes in OA, including CHI3L1, TSPYL2, TLR6, TIMP4, and NTRK2 (Fig. 6A). Further, qRT-PCR was used to examine whether miR-92b-5p regulated the expression of these potential target genes, and found that miR-92b-5p only negatively regulated TIMP4 expression, but did not affect the expression of other genes (Fig. 6B). Additionally, RIP experiment depicted both miR-92b-5p and TIMP4 were specifically enriched in anti-AGO2 antibody, but not in anti-IgG, suggesting that TIMP4 may be the target gene of miR-92b-5p (Fig. 6C). To confirm the above inference, the luciferase reporter assay was applied to analyze whether miR-92b-5p directly interacts with TIMP4. The results showed that the overexpression of miR-92b-5p apparently decreased the luciferase activity of TIMP4-WT, but did not affect the luciferase activity of TIMP4-Mut (Fig. 6D). Meanwhile, we examined the expression levels of endogenous miR-92b-5p and TIMP4 by qRT-PCR, and found that miR-92b-5p inhibition partially weakened the attenuating effect of LPS on TIMP4 expression in chondrocyte (Fig. 6E). In summary, these data demonstrated that TIMP4 was a direct target of miR-92b-5p.

Now that we have confirmed that TIMP4 is a target of miR-92b-5p, we would like to further analyze the impact of TIMP4 and miR-92b-5p interactions on cellular phenotype. Chondrocyte infected with miR-92b-5p inhibitor alone or in combination with shTIMP4 were treated with LPS for 48 h, and we examined the proliferation, apoptosis and ECM degradation capacity of chondrocytes. CCK-8 assay attested that miR-92b-5p inhibition obviously promoted proliferation in LPS-induced OA chondrocyte, and this effect was reversed by TIMP4 knockdown (Fig. 6F). Flow cytometry results show that miR-92b-5p inhibition abrogated the proapoptotic effect of LPS in chondrocytes, and this effect was attenuated by TIMP4 knockdown (Fig. 6G). Furthermore, western blotting indicated that miR-92b-5p inhibition attenuated the ECM degradation in LPS-induced OA chondrocytes, whereas TIMP4 knockdown partly reversed the attenuating effect of miR-92b-5p inhibition on chondrocyte ECM degradation (Fig. 6H). Collectively, the above data revealed that TIMP4 inhibition reversed the effects of miR-92b-5p knockdown on proliferation, apoptosis, and ECM degradation in LPS-induced OA chondrocyte.

### WTAP knockdown inhibited LPS-induced OA chondrocyte damage by regulating miR-92b-5p/TIMP4 axis

**Fig. 7:**
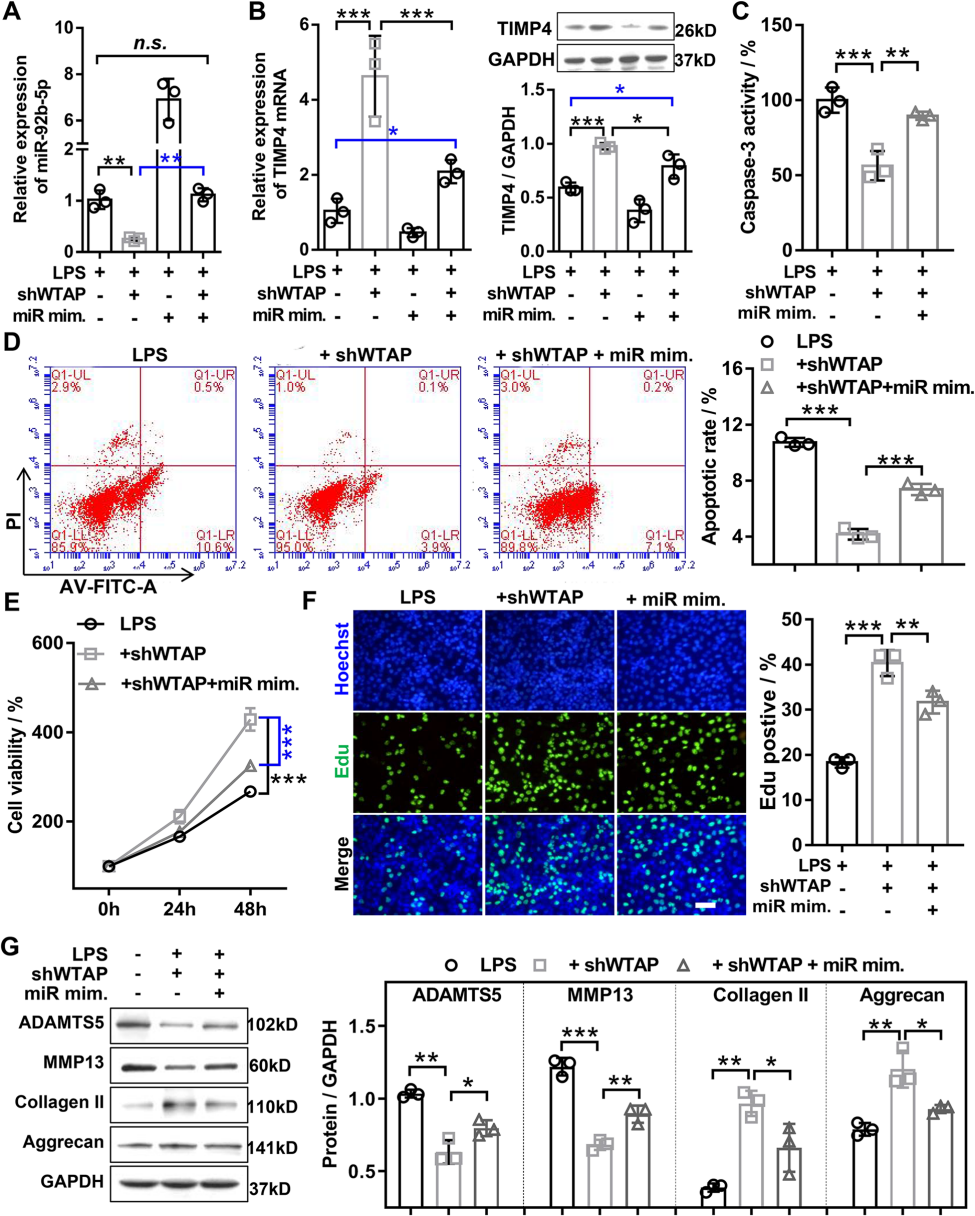
WTAP knockdown inhibited LPS-induced OA chondrocyte damage by regulating miR-92b-5p. Chondrocyte was infected with WTAP knockdown adenovirus alone or in combination with miR-92b-5p mimics, and then treated with 40 ng/mL LPS for 48 h. **A** The expression of miR-92b-5p was assessed by qRT-PCR. **B** The mRNA and protein expression levels of TIMP4 were measured by both qRT-PCR and western blotting. **C** The caspase-3 activity was evaluated by the caspase-3 activity kit. **D** The apoptosis of chondrocyte was evaluated by flow cytometry. **E** CCK-8 assay was used to detect the viability of chondrocyte. **F** EdU staining was used to assess the proliferation of chondrocyte. **G** Western blotting was applied to measure the protein expression levels of MMP13, ADAMTS5, Aggrecan, and Collagen II. LPS, lipopolysaccharide; miR mim., miR-92b-5p mimics; shWTAP, WTAP knockdown adenovirus. *N* = 3. ^*^P < 0.05, ^**^P < 0.01, and.^***^P < 0.001

To further confirm the biological function of the WTAP/miR-92b-5p/TIMP4 axis in OA, rescue experiments were employed. The results of qRT-PCR disclosed that WTAP knockdown significantly decreased miR-92b-5p expression in LPS-induced OA chondrocyte, and the effect was reversed by miR-92b-5p mimics (Fig. 7A). Meanwhile, WTAP knockdown resulted in significantly increased the mRNA and protein expression of TIMP4 in LPS-induced OA chondrocyte, but the above effect was only partially reversed by miR-92b-5p overexpression (Fig. 7B), suggesting that WTAP knockdown partially increased TIMP4 expression by inhibiting miR-92b-5p in LPS-induced OA chondrocyte.

**Fig. 8:**
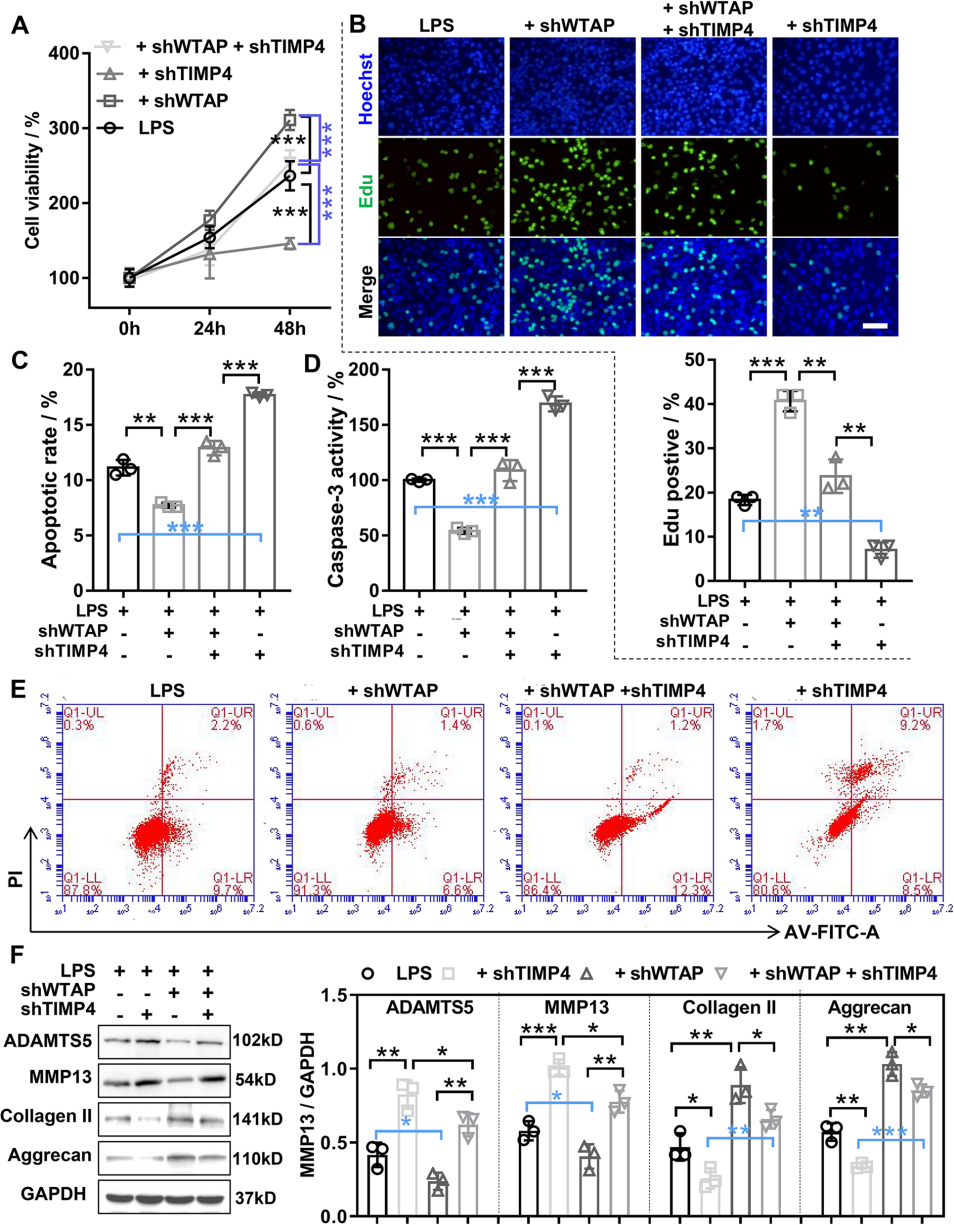
WTAP knockdown inhibited LPS-induced OA chondrocyte damage by upregulating TIMP4. Chondrocyte was infected with WTAP knockdown adenovirus alone or in combination with TIMP4 knockdown adenovirus, and then treated with 40 ng/mL LPS for 48 h. **A** CCK-8 was detected the viability of chondrocyte. **B** EdU staining was applied to detect the proliferation of chondrocyte. **C** and **E** The apoptosis of chondrocyte was assessed by flow cytometry. **D** The caspase-3 activity was measured by the caspase-3 activity kit. **F** Western blotting was applied to assess the protein expression levels of MMP13, ADAMTS5, Aggrecan, and Collagen II. LPS, lipopolysaccharide; shTIMP4, TIMP4 knockdown adenovirus; shWTAP, WTAP knockdown adenovirus. *N* = 3. ^*^P < 0.05, ^**^P < 0.01, and.^***^P < 0.001

To further confirm that the WTAP/miR-92b-5p axis regulates the biological function of chondrocyte, a series of rescue experiments were performed. The results of caspase-3 activity assay and flow cytometry assay attested that miR-92b-5p overexpression could partially weaken the decreased apoptosis in LPS-induced OA chondrocyte mediated by WTAP knockdown (Fig. 7C and D). The results of CCK-8 and EDU assay unmasked that miR-92b-5p overexpression could partially impaired the increased proliferation in LPS-induced OA chondrocyte mediated by WTAP knockdown (Fig. 7E and F). The results of western blotting indicated that miR-92b-5p overexpression could partially reverse the reduced ECM degradation in LPS-treated chondrocyte mediated by WTAP knockdown (Fig. 7G). Next, we verified that WTAP knockdown inhibited LPS-induced OA chondrocyte damage by increasing TIMP4. The results of CCK-8 and EdU disclosed that WTAP knockdown significantly enhanced the proliferation ability in LPS-induced OA chondrocytes, and this effect was reversed by TIMP4 knockdown (Fig. 8A and B). The results of flow cytometry and caspase-3 activity experiments showed that WTAP knockdown significantly reduced the LPS-induced apoptosis in OA chondrocyte, and this effect was reversed by TIMP4 knockdown (Fig. 8C, D, and E). In addition, western blotting demonstrated that WTAP knockdown significantly restrained the LPS-induced ECM degradation in OA chondrocyte, and this effect was impaired by TIMP4 knockdown (Fig. 8F). Taken together, our data demonstrated that WTAP knockdown inhibited LPS-induced OA chondrocyte damage by mediating the miR-92b-5p/TIMP4 axis.

### WTAP promoted TIMP4 mRNA degradation in OA chondrocyte in a m6A YTHDF2-dependent manner

**Fig. 9:**
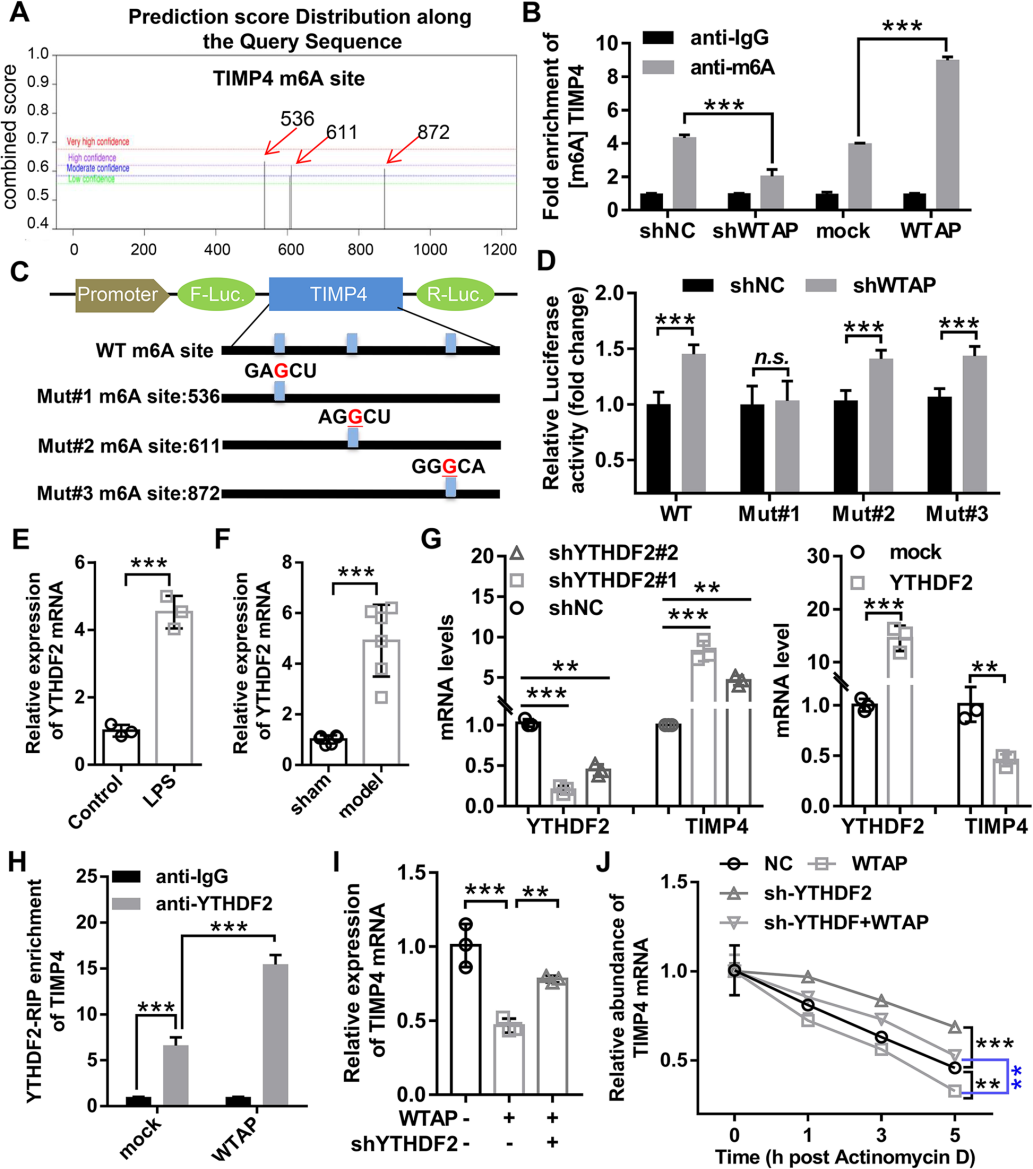
WTAP promoted TIMP4 mRNA degradation in a m6A YTHDF2-dependent manner. **A** The m^6^A modification sites on TIMP4 mRNA sequence were predicted by SRAMP, and found that there were 3 greater than moderate confidence m6A sites in TIMP4, including one high confidence m6A site and two moderate m6A methylation sites. **B** Chondrocyte was infected with shWTAP, shNC, mock, or WTAP, and then the m6A levels of TIMP4 was evaluated using MeRIP coupled with qRT-PCR. **C** The wild-type or mutant luciferase plasmids of TIMP4 m^6^A were cloned in pmirGLO. The mutant ones obtained A-G mutations on m6A motifs. **D** The luciferase reporter assay was used to detect the relative activity of the TIMP4- m^6^A-WT or TIMP4- m^6^A-Mut luciferase reporters in WTAP-knockdown SW1353 cells. **E** The mRNA expression of YTHDF2 in chondrocyte with or without LPS was detected by qRT-PCR. **F** The mRNA expression of YTHDF2 in mice OA cartilage tissue and mice normal cartilage tissue was detected by qRT-PCR. **G** The mRNA expression of YTHDF2 and TIMP4 was detected by qRT-PCR in chondrocyte infected with WTAP overexpressing or knockdown adenovirus. **H** RNA immunoprecipitation (anti-YTHDF2) in mock or WTAP-overexpression cells was conducted followed by RT-qPCR to measure the amount of TIMP4 mRNA binding to YTHDF2. **I** Chondrocyte was infected with WTAP overexpression adenovirus alone or in combination with YTHDF2 knockdown adenovirus, the mRNA expression of TIMP4 was determined by qRT-PCR. **J** Chondrocyte were infected with WTAP overexpression adenovirus alone or in combination with YTHDF2 knockdown adenovirus, the RNA decay rate was measured in chondrocyte treated with Actinomycin D. shWTAP, WTAP knockdown adenovirus; WTAP, WTAP overexpression adenovirus; shYTHDF2, YTHDF2 knockdown adenovirus; YTHDF2, YTHDF2 overexpression adenovirus. *N* = 3 ~ 6. ^*n.s*^*P* < 0.01, ^**^P < 0.01, and.^***^P < 0.001

Previous studies showed that WTAP could only partially regulate TIMP4 expression through miR-92b-5p (Fig. 7B). Therefore, we speculated that there may be other mechanisms by which WTAP negatively regulated TIMP4 expression in OA. Previous studies have shown that WTAP mediates m6A modification of target genes to suppress the mRNA expression of target genes [21, 22]. We predicted m^6^A modification sites on TIMP4 mRNA sequences using SRAMP, and found that there were 3 greater than moderate confidence m6A sites in TIMP4, including 1 high confidence m6A site and 2 moderate m6A methylation sites (Fig. 9A). Therefore, we speculated that WTAP might promote TIMP4 degradation by mediating TIMP4 m6A modification. MeRIP coupled with qRT-PCR demonstrated that WTAP knockdown dramatically decreased TIMP4 m6A levels in LPS-induced OA chondrocyte, while overexpression of WTAP displayed contrary effects (Fig. 9B), suggesting that WTAP regulated TIMP4 m6A levels in LPS-induced OA chondrocyte. To further demonstrate the function of WTAP-mediated m^6^A modification on TIMP4, we performed the luciferase reporter assay. The luciferase reporter gene contains wild-type (WT) and three mutant (Mut) plasmids, where the Mut reporter is bases (A) into bases (G) in the predicted m^6^A sites to eliminate the TIMP4 m6A modification and WT reporter contains all m^6^A sites of TIMP4 (Fig. 9C). The results demonstrated that the luciferase activity of TIMP4-m6A-WT, TIMP4-m6A-MUT#2, and TIMP4-m6A-MUT#3 moderately intensified by WTAP knockdown in SW1353, but the luciferase activity of TIMP4-m6A-MUT#1 did not affected by WTAP knockdown (Fig. 9D), suggesting that WTAP regulated TIMP4 expression by mediating m6A site #1 rather than m6A site #2 and #3. YTH domain family protein 2 (YTHDF2) is a well-know m^6^A reader and frequently participates in the regulation of mRNA degradation by reading the m6A modification [23]. Figure 1A showed that YTHDF2 was upregulated in OA cartilage tissue compared to normal cartilage tissue. Meanwhile, we found that YTHDF2 was highly expressed in OA model in vitro and in vivo (Fig. 9E and F). Besides, qRT-PCR results demonstrated that TIMP4 mRNA levels were sharply increased in YTHDF2-depleted OA chondrocyte and decreased in YTHDF2- overexpressed OA chondrocyte (Fig. 9G). And the results of RIP-qPCR uncovered that YTHDF2-specific antibody dramatically enriched TIMP4 mRNA compared with the IgG antibody, while overexpression of WTAP remarkably elevated the enrichment of TIMP4 mRNA (Fig. 9H), revealing that overexpression of WTAP increased the interaction between YTHDF2 and TIMP4. Moreover, the reduction of TIMP4 induced by overexpression of WTAP could be retrieved by inhibition of YTHDF2 in OA chondrocyte (Fig. 9I). Meanwhile, overexpression of WTAP would shorten the half-life of TIMP4 RNA, and this effect was reversed by YTHDF2 knockdown (Fig. 9J). Collectively, the above evidence suggested that WTAP mediated TIMP4 mRNA degradation in a m6A YTHDF2-dependent manner.

## Discussion

As ubiquitous transcriptome modifications in most eukaryotic mRNAs, catalytic enzymes affecting m6A levels have been extensively reported [24]. METTL3, METTL4, and WTAP are the important components of methyltransferase. Among them, METTL3 serves as the S-adenosylmethionine (SAM) binding subunit, METTL14 serves as an RNA-binding scaffold for substrate recognition, and WTAP is essential for stabilizing METTL3 and METTL14 [25, 26]. METTL3 was reported to accelerate IL-1β-induced chondrocyte damage by regulating m6A-mediated miR-126-5p maturation [27], indicating that m6A can participate in and regulate the progression of OA. In this study, our data revealed for the first time that WTAP significantly up-regulated and accompanied by an increase in m6A levels in OA cartilage tissue and chondrocyte, suggesting that WTAP might be involved in OA progression as an m6A mediator.

At present, WTAP has mostly been demonstrated to be involved in cell cycle regulation [28], X chromosome inactivation [29], tumor development [30, 31] and alternative splicing [32]. However, very few studies of WTAP in OA have been reported. To further explore the biological function of WTAP in OA, we performed both cellular and animal level studies. During cartilage injury and remodeling, chondrocyte plays a role in maintaining homeostasis, and their proliferation and apoptosis are closely related to the occurrence and development of OA [33]. Therefore, increasing chondrocyte activity and inhibiting chondrocyte apoptosis may be therapeutic strategies to inhibit the progression of OA. In the present study, we disclosed that WTAP knockdown evidently promoted proliferation and inhibited apoptosis in LPS-induced OA chondrocytes, while WTAP overexpression resulted in opposite phenotypes. Poor OA microenvironment is associated with chondrocyte damage [34]. Therefore, improving the microenvironment can help alleviate OA progression. In our study, we found that WTAP deletion remarkably suppressed LPS-induced pathological changes in cartilage tissue. During OA development, inadequate ECM synthesis capacity leads to progressive cartilage degeneration, which reduced chondrocyte support [35]. Here, the protein data depicted that WTAP deficiency significantly promoted ECM synthesis and inhibited ECM degradation, manifested as an increased protein expression of collagen II and Aggrecan, and a decreased protein expression of MMP13 and ADAMTS5, which was consistent with Liu et al.'s report that shMETTL3 inhibited ECM degradation [36]. Taken together, the above findings suggested that shWTAP may act as an m6A mediator to alleviate OA.

Using miRNA-Seq and MeRIP, we identified the upregulated miR-92b-5p in OA as a downstream target for WTAP-mediated m6A modification. Previous studies have shown that miRNAs contain m6A sites in their pri-miRNAs and m6A modification plays a facilitating role in miRNA maturation [37, 38]. Fortunately, three m6A sites were found on the pri-miR-92b sequence. Recently, Alarcon et al. proved that m6A modification can be mediated by METTL3 to tag pri-miRNAs and process their maturation [39]. Also in our study, we examined the expression of pri-miR-92b, pre-miR-92b, and miR-92b-5p in WTAP-depleted cells, and revealed that pri-miR-92b was significantly elevated in WTAP-depleted cells, while pre-miR-92b and miR-92b-5p were significantly decreased, as reduced m6A levels would lead to a stall in pri-miRNA processing [24]. In WTAP-overexpressing cells, opposite expression trends were found for the aforementioned genes. Moreover, MeRIP assay, dual luciferase reporter assay, and qRT-PCR in WTAP knockdown chondrocyte further supported this finding. Cells recognized and processed pri-miRNAs to form miRNAs with the help of microprocessor complexes, including DROSHA and DGCR8 [40]. DGCR8, as the main binding protein of DROSHA, can bind to pri-miRNAs through the two double-stranded RNA binding regions at its C-terminal, recruiting and guiding DROSHA to shear at the correct position of the pri-miRNAs to generate pre-miRNAs [41]. Alarcón et al. proved that METTL3 knockdown inhibited DGCR8 binding to pri-miRNAs, resulting in an overall decrease of mature miRNAs and a concomitant accumulation of unprocessed pri-miRNAs [39]. Consistently, in our study, we found that pri-miR-92b was found slightly accumulated, while pre-miR-92b and miR-92b-5p were found remarkably reduced in pri-miR-92b [m6A]-Mut. Collectively, miR-92b-5p was regulated and processed to mature in an m6A-dependent manner as a downstream target of WTAP.

MiRNA degrades mRNAs or blocks their translation by base-pairing with mRNA-directed silencing complexes of target genes, and available data indicate that more than 50% of RNA molecules are controlled by miRNAs [42]. Therefore, elucidating the molecular mechanisms of miRNAs in the occurrence of OA will be useful for the treatment and diagnosis of OA. Our study demonstrated that TIMP4 was a direct target of miR-92b-5p through bioinformatics prediction and molecular biology. TIMP4, belonging to the TIMPs family, specifically restrains the activity of MMPs, maintains homeostasis in healthy cartilage, and is a naturally occurring inhibitor of MMP and ADAMTS function [43]. Chen et al. have shown that the expression of TIMP4 is reduced in OA chondrocyteand was participates in the MMPs/TIMP balance of cartilage [44]. Interestingly, our data showed that TIMP4 was significantly downregulated after miR-92b-5p overexpression. At the same time, miR-92b-5p inhibition was found to significantly promote chondrocyte activity and ECM synthesis as well as remarkably inhibited apoptosis and ECM degradation. Furthermore, we demonstrated that WTAP knockdown inhibited OA deterioration by regulating miR-92b-5p/TIMP4 axis.

Our previous study showed that WTAP could only partially regulate TIMP4 expression through miR-92b-5p. Therefore, we speculated that there may be other mechanisms by which WTAP negatively regulated TIMP4 expression in OA. Here, we observed that WTAP regulated TIMP4 m6A levels and suppressed TIMP4 expression. Accumulating data have suggested that m6A modification is directly related to mRNA stability. For m6A modification to perform its function, it must first be recognized by the m6A reader protein [45]. YTHDF2 is the first m6A reader protein identified and affect the stability of m6A-modified RNAs by targeting them to mRNA decay sites [46]. Chen et al. Have reported that WTAP regulates m^6^A-mediated HMBOX1 expression in a YTHDF2-independent manner [22]. Therefore, we assumed that WTAP mediated TIMP4 mRNA degradation in a YTHDF2-independent manner. As expected, we found that WTAP overexpression increased the binding of YTHDF2 to TIMPT mRNA. Meanwhile, we found that overexpression of WTAP could shorten the half-life of TIMP4 mRNA, and this effect was reversed by YTHDF2 knockdown. Collectively, the above evidence suggested that WTAP mediated TIMP4 mRNA degradation through YTHDF2-dependent manner.

## Conclusion

In summary, our data revealed that WTAP knockdown alleviated OA progression via modulation of the miR-92b-5p/TIMP4 axis in a m6A-dependent manner, highlighting WTAP as a potentially promising preventive and therapeutic target for OA.

### Supplementary Information

**Additional file 1: Fig. S1.** The expression levels of miR-92b-5p were detected by qRT-PCR.

## Data Availability

All data are presented in this paper.
