# Supplementary material for: N6-methyladenosine (m6A) methyltransferase WTAP-mediated miR-92b-5p accelerates osteoarthritis progression
Source: Cell Commun Signal. 2023 Aug 10;21:199. doi: 10.1186/s12964-023-01228-8 (PMC10416510; doi:10.1186/s12964-023-01228-8)
Supplement: Supplementary file 2 — Additional file 1: Fig. S1. The expression levels of miR-92b-5p were detected by qRT-PCR. [file 12964_2023_1228_MOESM1_ESM.docx]

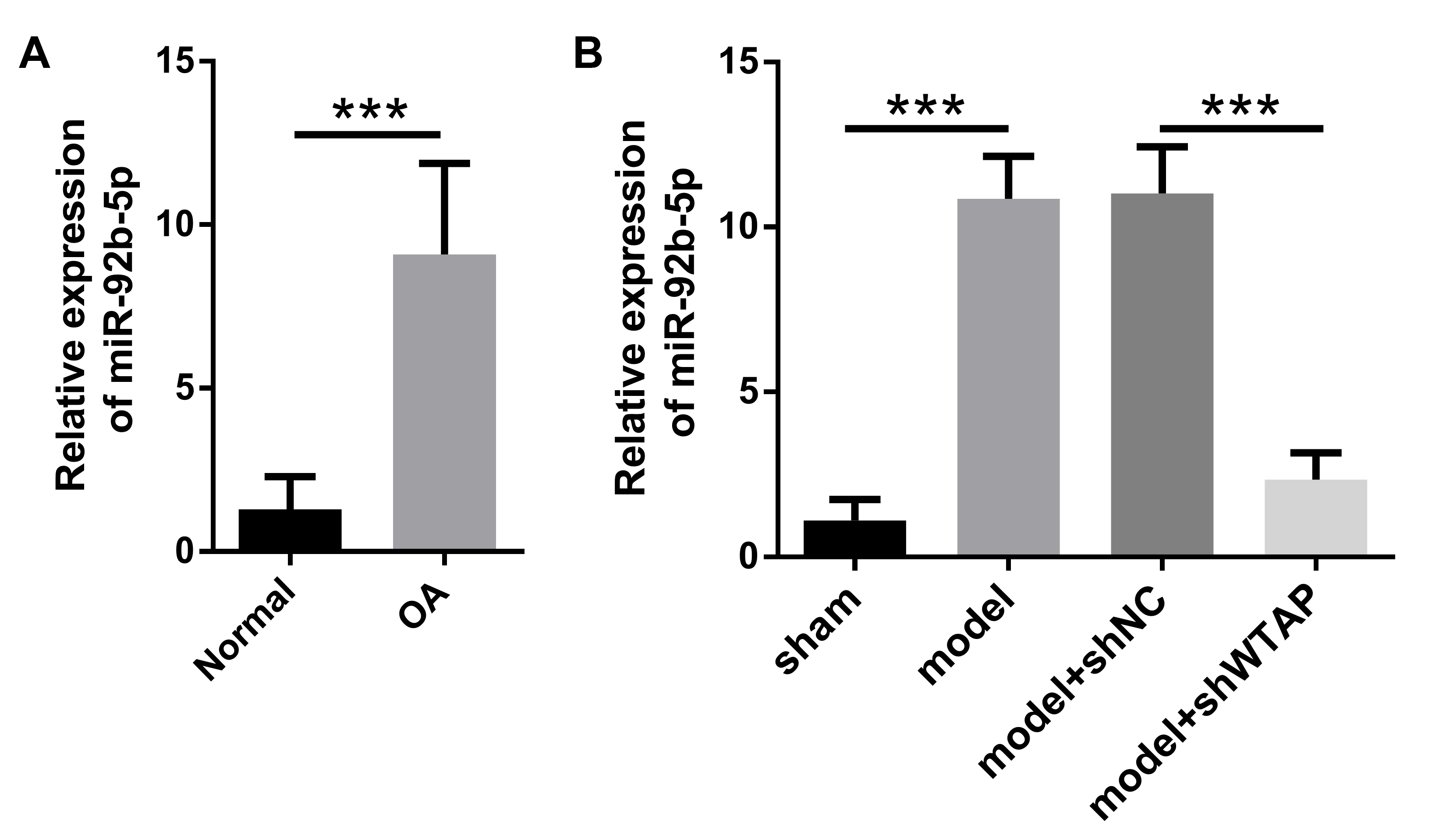


**Fig. S1 The expression levels of miR-92b-5p were detected by qRT-PCR.** (A) The qRT-PCR was used to measure the expression levels of miR-92b-5p in cartilage tissue of patients with or without OA. Normal, cartilage tissue of patients without OA; OA, cartilage tissue of patients with OA. (B) RT-qPCR was employed to assess the impact of WTAP knockdown on the expression of miR-92b-5p in cartilage tissue of OA mice. Sham, mice without destabilizing the medial meniscus (DMM); model, DMM surgery-induced OA mice; AAV-shNC, negative control corresponding to AAV-shWTAP; AAV- shWTAP, WTAP knockdown adeno-associated virus. N = 6. ^***^*P*<0.001.
